# A *DSPP* Mutation Causing Dentinogenesis Imperfecta and Characterization of the Mutational Effect

**DOI:** 10.1155/2013/948181

**Published:** 2012-12-27

**Authors:** Sook-Kyung Lee, Kyung-Eun Lee, Su Jeong Song, Hong-Keun Hyun, Sang-Hoon Lee, Jung-Wook Kim

**Affiliations:** ^1^Department of Pediatric Dentistry and Dental Research Institute, School of Dentistry, Seoul National University, 275-1 Yongon-dong, Chongno-gu, Seoul 110-768, Republic of Korea; ^2^Department of Molecular Genetics and Dental Research Institute, School of Dentistry, Seoul National University, 275-1 Yongon-dong, Chongno-gu, Seoul 110-768, Republic of Korea

## Abstract

Mutations in the *DSPP* gene have been identified in nonsyndromic hereditary dentin defects, but the genotype-phenotype correlations are not fully understood. Recently, it has been demonstrated that the mutations of *DSPP* affecting the IPV leader sequence result in mutant DSPP retention in rough endoplasmic reticulum (ER). In this study, we identified a Korean family with dentinogenesis imperfecta type III. To identify the disease causing mutation in this family, we performed mutational analysis based on candidate gene sequencing. Exons and exon-intron boundaries of *DSPP* gene were sequenced, and the effects of the identified mutation on the pre-mRNA splicing and protein secretion were investigated. Candidate gene sequencing revealed a mutation (c.50C > T, p.P17L) in exon 2 of the *DSPP* gene. The splicing assay showed that the mutation did not influence pre-mRNA splicing. However, the mutation interfered with protein secretion and resulted in the mutant protein remaining largely in the ER. These results suggest that the mutation affects ER-to-Golgi apparatus export and results in the reduction of secreted DSPP and ER overload. This may induce cell stress and damage processing and/or transport of dentin matrix proteins or other critical proteins.

## 1. Introduction

Hereditary dentin defects are categorized into three types of dentinogenesis imperfecta (DGI) and two types of dentin dysplasia (DD) [1]. DGI type I (MIM 166240) is a syndromic dental phenotype of osteogenesis imperfecta. The phenotype is similar to DGI type II but the penetrance is incomplete and the expressivity is also variable [1]. DGI type II (MIM 125490) is characterized by opalescent discolored dentition, pulpal calcification, and bulbous crown shape. DGI type III (MIM 125500), originally thought to be specific to the Brandywine isolate, is a severe form of DGI type II with multiple pulp exposures and shell-like teeth [2]. DD type II (MIM 125420) is similar to DGI type II in the deciduous dentition but tooth discoloration is minimal, and pulp chambers are thistle-tube shaped with pulp stones in the permanent dentition. In DD type I (MIM 125400), teeth are normal in shape, form, and consistency in the deciduous and permanent dentition. In some cases, color of the teeth may display a slight amber discoloration. However, the roots are short and the pulp obliteration results in a crescent-shaped pulpal remnant in the permanent dentition and a total pulpal obliteration in the deciduous dentition [1].

 The dentin sialophosphoprotein (*DSPP*) gene is located on chromosome 4q21 and encodes the major non-collagenous protein in the dentin matrix [2]. DSPP is rapidly cleaved by proteases into three major proteins: dentin sialoprotein (DSP), dentin glycoprotein (DGP), and dentin phosphoprotein (DPP) [3, 4]. Mutations in the *DSPP* gene have been identified to cause DGI type II, III, and DD type II [5–25]. Therefore, these diseases are not separate, but are allelic with differing severity [2]. Even though there are several other candidate genes for nonsyndromic hereditary dentin defects [26], only mutations in the *DSPP* gene have been identified to date.

 Reduction of DSPP and/or improper mineralization induced by the mutant *DSPP* gene could result in defective dentin mineralization. Alternatively, accumulation of the mutant DSPP in the odontoblast would result in cellular damage and influence protein processing and/or transporting system during rapid dentin matrix formation, particularly for dentin matrix proteins including DSPP [21].

A functional domain at the N-terminus of DSPP and its possible role in the signal peptide cleavage have been suggested due to its proximity to the signal peptide cleavage site [21]. Secreted DSPP has a highly conserved N-terminal sequence beginning with Ile Pro Val (IPV) after cleavage of the 15 amino acid signal peptide. Mutations affecting the IPV domain would result in errors in signal peptide cleavage processing and subsequently affect protein secretion.

In this study, we identified a mutation in the *DSPP* gene that causes DGI type III and investigated the effect of the mutation on pre-mRNA splicing and protein secretion in order to better understand the molecular genetic pathogenesis underlying the aberrant dentin biomineralization.

## 2. Materials and Methods

### 2.1. Enrollment of Human Subjects

The protocols and subject consents for this study were reviewed and approved by the Institutional Review Board at Seoul National University Dental Hospital. We identified a Korean family with dentinogenesis imperfecta type III, and seven family members were recruited for this study. Clinical and radiologic examinations were performed, and blood samples were collected with the understanding and written consent of each subject according to the Declaration of Helsinki. 

### 2.2. Primers, Polymerase Chain Reaction (PCR), and DNA Sequencing

Genomic DNA was extracted from peripheral whole blood using the QuickGene DNA whole blood kit S with QuickGene-Mini80 equipment (Fujifilm, Tokyo, Japan) according to the manufacturer's instructions. Primers and conditions for PCR and DNA sequencing were previously described [10]. PCR reactions were performed using HiPi DNA polymerase premix (ElpisBio, Daejeon, Korea), and the PCR products were purified with a PCR Purification Kit (ElpisBio). DNA sequencing was performed at the DNA sequencing center (Macrogen, Seoul, Korea). All nucleotide numbering began from the A of the ATG translational initiation codon of the human *DSPP* reference sequence (NM-014208.3).

### 2.3. Cloning of Genomic DNA and Mutagenesis

A wildtype human *DSPP* gene fragment including exons two, three, and four was cloned into the pSPL3 splicing vector as previously described [13]. Mutation was introduced by PCR mutagenesis (forward: 5′-GTA GCA TGG GCC ATT C**T**A GTA AGT ATG CCT TTC-3′, reverse: 5′-GAA AGG CAT ACT TAC T**A**G AAT GGC CCA TGC TAC-3′), and the sequence was confirmed by direct sequencing.

### 2.4. *In Silico* Splicing Assay

The mutational effect on pre-mRNA splicing was analyzed by two computer programs: SplicePort (http://spliceport.cbcb.umd.edu/) and NNSPLICE version 0.9 (http://www.fruitfly.org/seq_tools/splice.html).

### 2.5. *In Vitro* Splicing Assay

COS-7 cells were transfected with wildtype and mutant pSPL3 vectors, and total RNA was isolated after 48 hours using TRIzol reagent (Invitrogen, Carlsbad, CA, USA). cDNA was generated from 4 *μ*g RNA using RT-PCR premix (ElpisBio). RT-PCR amplification (wild type amplicon size: 666 bp; forward: 5′-TAG AGT CGA CCC AGC ACC AT-3′, reverse: 5′-CCT CGT TTC TAC AGG AAT TCT CA-3′) was performed using HiPi PCR premix (ElpisBio). Amplification products were resolved on a 1% agarose gel and characterized by DNA sequencing.

### 2.6. DSP Secretion Analysis

The coding region of the entire human DSP protein (from aa 1 to aa 382) was amplified by an RT-PCR reaction using PCR primers (forward: 5′-GGA AGC TTG AAA ATG AAG ATA ATT ACA TA-3′, reverse: 5′-GGG GAT CCC CGC TGG GAC CCT TGA TTT CTA-3′) and cloned into a pEGFP-N1 vector after double digestion with *Hind*III and *Bam*HI restriction endonucleases. PCR mutagenesis was performed to replace C with T (c.50C > T) using primers (forward: 5′-GCA GTA GCA TGG GCC ATT C**T**A GTT CCT CAA AGC-3′, reverse: 5′-GCT TTG AGG AAC T**A**G AAT GGC CCA TGC TAC TGC-3′). Sequences of normal and mutant pEGFP-N1 vectors were confirmed by direct plasmid sequencing. Normal and mutant DSP pEGFP-N1 vectors were transiently transfected into HEK293T cells using Lipofectamine 2000 reagent (Invitrogen, Carlsbad, CA, USA). Cell lysate and culture media (Dulbecco's Modified Eagle's Media) without serum were harvested after 48 hours. Cell lysate (40 *μ*g) and culture media (20 *μ*L) were run on a 10% SDS-polyacrylamide gel and subjected to Western blot using anti-DSP (Santa Cruz Biotechnology, Santa Cruz, CA, USA) and anti-GAPDH (ABM, Richmond, ON, Canada) antibodies. Silver staining was performed to normalize the amount of the culture media. Band intensity was measured using ImageJ (NIH). Experiments were duplicated, and statistical analysis (independent *t*-test) was performed to compare the amount of wildtype and mutant DSP in the media and cell lysate.

### 2.7. Fluorescent Immunocytochemistry

Normal and mutant DSP pEGFP-N1 vectors were transiently transfected into HEK293T cells using lipofectamine 2000 reagent (Invitrogen). The transfected cells were seeded on the cover glass coated with poly-L-lysine (Sigma-Aldrich, St. Louis, MO, USA). After 24 hours, cells were washed with PBS and fixed with 4% paraformaldehyde for 20 min. Permeabilized cells were treated with 0.1% Triton X-100 for 15 min and subsequently blocked with 10% goat serum (Vector Lab, Burlingame, CA, USA) for 1 hr. For ER staining, slides were incubated with mouse monoclonal anti-Calnexin (CANX) antibody (Millipore, Milford, MA, USA) for 2 hr followed by incubation with Texas Red conjugated goat anti-mouse secondary antibody (Santa Cruz Biotechnology) for 1 hr. For Golgi apparatus staining, the slides were incubated with rabbit monoclonal anti-Golgi matrix protein (GM130) antibody (Epitomics, Burlingame, CA, USA) for 2 hr followed by incubation with Texas Red conjugated goat anti-rabbit secondary antibody (Vector Lab) for 1 hr. Nuclei were stained with 20 *μ*M H33342 (Sigma-Aldrich) for 10 min. Slides were coverslipped with fluorescence mounting medium (Dako, Carpinteria, CA, USA) and the fluorescent images were captured using an Olympus FV300 laser-scanning confocal microscope.

## 3. Results and Discussion

### 3.1. Clinical Findings

**Figure 1:**
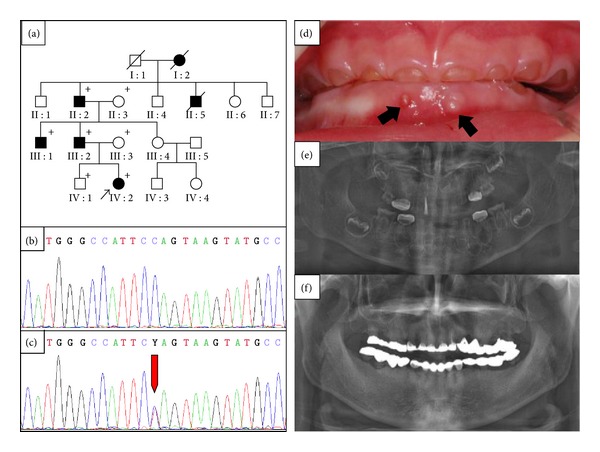
Pedigree, mutational analysis, and clinical photos. (a) Pedigree of the proband's family. The “plus” symbol indicates members recruited for this study. (b) and (c) DNA sequencing chromatogram of a normal control and an affected individual. The red arrow indicates the mutated nucleotide (c.50C > T). (d) Frontal clinical photo of the proband. The deciduous dentition exhibits severe attrition and marked discoloration. Periapical abscesses are indicated by black arrows. (e) Panoramic radiogram of the proband during treatment at 2.8 yrs of age. The mandibular primary central incisors were extracted, and all primary first molars were treated with stainless steel crowns. Pulp treatment was performed for the maxillary primary lateral incisor, and wide pulp chambers can be identified in the remaining anterior teeth. (f) panoramic radiogram of an affected individual at 29.7 yrs of age (III : 2).

The proband (IV : 2) first visited the Pediatric Dental Clinic of the Seoul National University Dental Hospital at 2.5 years of age (Figure 1(a)). Her deciduous dentition displayed severe attrition and marked discoloration. Periapical inflammations were observed in several teeth in which the pulp tissue was exposed by attrition (Figure 1(d)). Her father (III : 2) was 29 years old, but had lost many teeth and had full-coverage prosthodontics on his remaining permanent teeth (Figure 1(e)). There was no history of bone fragility or symptoms of hearing loss in the family.

### 3.2. Mutation Results

Sequencing analysis revealed a C to T transition in exon 2 (c.50C > T) of the *DSPP *gene. This sequence variation correlated perfectly with the presence of the disease (Figure 1). This sequence variation was not present in 100 unaffected control individuals from the Korean population (data not shown). This suggests that the mutation is not a common variant of the *DSPP* gene. The same mutation has been recently identified in a Chinese family [25]. This mutation changes the proline residue at the +2-position (P2′) from the signal peptide cleavage site to the leucine residue (p.P17L). The Pro17 residue is conserved in human, mouse, rat, pig, and cow.

### 3.3. Splicing Assay

*In silico* analyses of the mutational effect on pre-mRNA splicing were inconsistent. One program yielded a change in the prediction value suggesting a defective splicing event (0.90 → 0.78 as a donor site), while the other program did not (0.98 → 0.98). 

**Figure 2:**
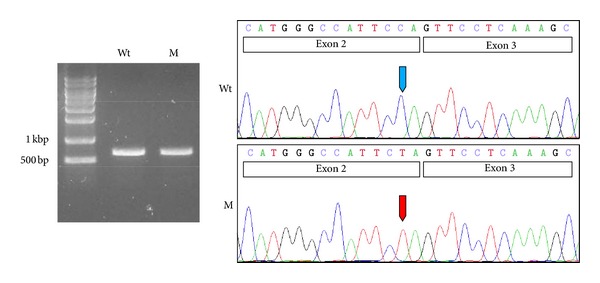
*In vitro* splicing assay. RT-PCR analyses of the wildtype (Wt) and mutated (M) pSPL3 vector. Both showed one normally spliced product that was confirmed by sequencing. The sizes of the marker band are shown in the gel image. Exons are shown in the box in the sequencing chromatogram. Wildtype and mutated nucleotide are indicated by blue and red arrows, respectively.

RT-PCR showed a single amplified product in both the wildtype and the mutant pSPL3 vectors (Figure 2). The forward primer binds at the boundary of the donor site of the pSPL3 vector and at the beginning of exon 2 of the *DSPP* gene, while the reverse primer binds at the middle of exon 4 of the *DSPP* gene. If intron 2 was not properly excised, the size of the amplicon would be 1811 bp. Sequencing analysis of the amplicons exhibited normally spliced sequences, indicating that this mutation in the second position from the end of exon 2 (c.50C > T) did not influence normal pre-mRNA splicing and resulted in pure missense mutations (p.P17L) (Figure 2).

### 3.4. DSP Secretion Analysis

**Figure 3:**
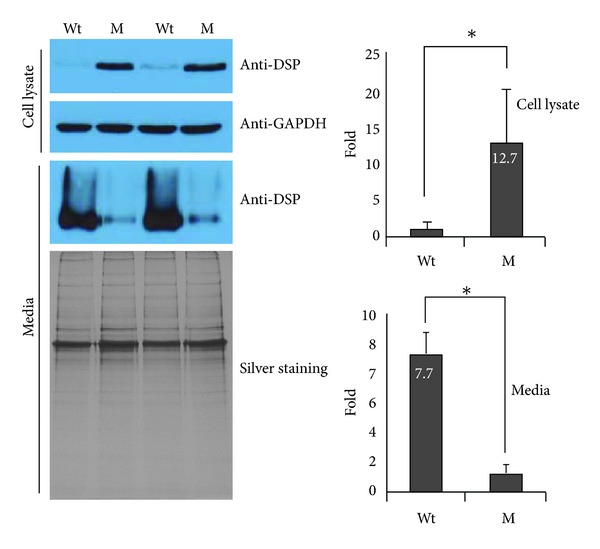
Protein expression analysis. Western blot analysis was performed to detect wildtype (Wt) and mutant (M) DSP in the cell lysate and culture media. The cell lysate was normalized with GAPDH. The secreted amount of wildtype DSP was more than 7-fold greater compared to the P17L mutant DSP (*P* < 0.01). Cellular retention of the mutant DSP was detected in the cell lysate. Western blot of the cell lysate showed that intracellular retention of the P17L mutant DSP was much higher (12.1 folds) than the wildtype DSP (*P* < 0.01). The asterisk indicates a statistically significant difference (*P* < 0.01). Fold increase values are shown in the bar.

Wildtype DSP was efficiently secreted to the culture media, while only a small amount of the mutant DSP was detected in the culture media. The secreted amount of wildtype DSP was more than 7-fold greater compared to the P17L mutant DSP (*P* < 0.01) (Figure 3). Cellular retention of the mutant DSP was detected in the cell lysate. Western blot of the cell lysate showed that intracellular retention of the P17L mutant DSP was much higher (12.1-fold) than the wildtype DSP (*P* < 0.01).

### 3.5. Fluorescent Immunocytochemistry

**Figure 4:**
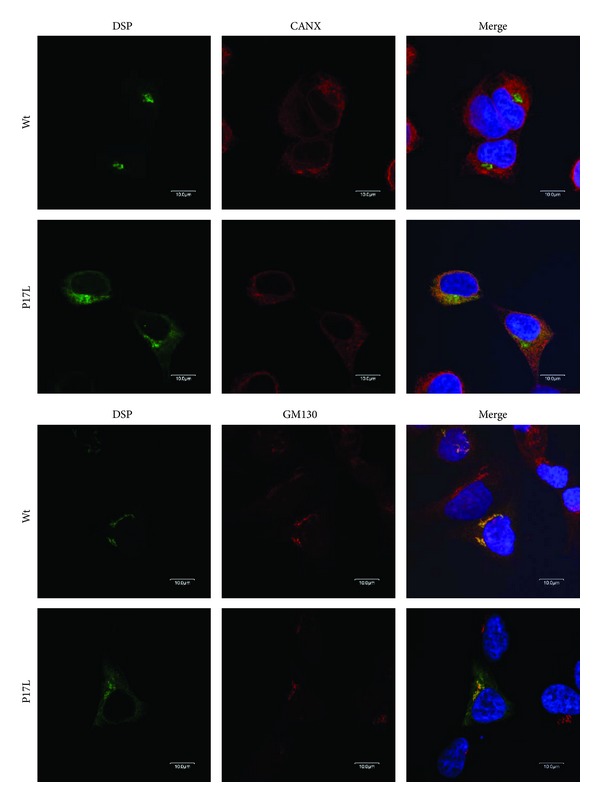
Fluorescent immunocytochemistry. Confocal laser-scanning images were captured to detect localization of the GFP-tagged wildtype (Wt) and mutant (P17L) DSP in HEK293T cells. Anti-Calnexin (CANX) antibody was used for ER staining, and anti-Golgi matrix protein (GM130) antibody was used for Golgi apparatus staining. Nuclei were stained with H33342. Wildtype DSP was localized exclusively in the Golgi apparatus. The mutant DSP largely remained in the ER, although a portion was localized in the Golgi apparatus.

Wildtype DSP was localized exclusively in the Golgi apparatus (Figure 4). In contrast, the P17L mutant DSP showed widespread expression in the cytoplasm. The mutant DSP largely remained in the ER, even though a portion was localized in the Golgi apparatus.

### 3.6. Discussion

The proband showed multiple pulp exposures and severe attrition. The father of the proband was 29 years old, but had lost many teeth. Interestingly, the disease causing the mutation of the Brandywine isolate was c.49C > T (p.P17S) [14]. The same mutation (c.49C > T, p.P17S) was also found in a Chinese family [15]. The clinical phenotype was also severe according to the clinical photos and panoramic radiograph. Pro17 is a well-conserved amino acid, and it is one of the mutational hot spots in the *DSPP* gene [25]. This data confirms that DGI type III is not confined to a specific (Brandywine isolate) population and is a severe form of DGI type II. Another mutation changing Pro17 (c.49C > A, p.P17T) was reported in a Chinese family [6]. Clinical severity was not reported in detail, but the affected individuals also presented with progressive sensorineural high-frequency hearing loss. However, hearing loss is not common in DGI families, so further studies are needed to characterize the functional role of *DSPP* mutations in the pathogenesis of hearing loss.

The identified mutation was located in the second nucleotide position from the end of exon 2. Despite its close proximity to the exon-intron boundary, the mutation did not influence normal pre-mRNA splicing. Therefore, the mutation resulted in a pure missense mutation and changed the highly conserved amino acid from Pro17 to Leu17.

The mutation could affect normal signal peptide cleavage based on its proximity to the cleavage site and the nature of the changed amino acid. Pro17 is located at the P2′ position from the signal peptide cleavage site (between Ala15 and Ile16). Proline is a nonpolar neutral amino acid with a hydropathy index of −1.6, while leucine is a non-polar neutral amino acid with a hydropathy index of 3.8. *In silico* analyses predicted reduced efficiency in the signal peptide cleavage. Changing Pro17 to Leu17 decreased the signal peptide probability (0.995 → 0.964) and the maximum cleavage site probability score (0.947 → 0.694) by SignalP 3.0 (http://www.cbs.dtu.dk/services/SignalP/). A decreased cleavage score (9.2 → 7.0) was also obtained by SIG-Pred (http://bmbpcu36.leeds.ac.uk/cgi-bin/sig_pred/Signal.cgi).

DSP expression and secretion were greatly affected by the introduction of the mutation. The amount of mutant DSP in the culture media was extremely small compared to the amount of wildtype DSP. In contrast to the culture media, mutant DSP was detected in the cell lysate, while wildtype DSP was faintly detected. These results indicate that the secretion of mutant DSP protein was severely affected and some mutant DSP protein was retained in the cell. This result is in accordance with the recent study on the rough ER trafficking errors caused by mutant DSPP [27].

Fluorescent immunocytochemical data showed that the wildtype DSP was efficiently transported to the Golgi apparatus for further export to the secretory pathway. However, the mutant DSP was largely retained in the ER, even though a portion was transported to the Golgi apparatus. Reduced efficiency in signal peptide cleavage could result in ER retention. However, this retention could also be resulted from disruption of the ER export signal by the Proline at the P2′ position [28].

A mutation (c.16T > G, p.Y6D) in the signal peptide resulted in DD type II, which is considered as a mild form of DGI type II. This mutation was shown to result in an absence of DSPP protein production from the mutated allele due to the defective signal peptide [8]. The amount of DSPP protein resulting from the mutated allele was nearly as absent as the mutation in this study. Therefore, the phenotypic difference cannot be explained only by the reduction in the amount of wildtype DSPP. The P17L mutant DSPP protein can be translocated into the ER with an intact signal peptide, but is expected to have a dominant-negative effect on cell function and/or on dentin matrix mineralization due to incomplete signal peptide cleavage [21], loss of the ER export signal, or protein misfolding. In addition to the defect in secretory pathway itself, some secreted mutant DSPP may cause defective mineralization. Further study is required to determine the exact cause of the pathogenesis of the DSPP-associated dentin defect.

## 4. Conclusion

In summary, we have identified a mutation in exon 2 of the *DSPP* gene that caused DGI type III. The mutation did not influence pre-mRNA splicing but caused ER retention and defective protein secretion. It is possible that the severe phenotype observed in the affected family is caused by a dominant negative effect. Future molecular studies on the effect of individual mutation and function of DSPP may provide better insight into the normal and pathological basis of dentin biomineralization.

## References

[1] Shields ED, Bixler D, El-Kafrawy AM (1973). A proposed classification for heritable human dentine defects with a description of a new entity. *Archives of Oral Biology*.

[2] Kim JW, Simmer JP (2007). Hereditary dentin defects. *Journal of Dental Research*.

[3] von Marschall Z, Fisher LW (2010). Dentin sialophosphoprotein (DSPP) is cleaved into its two natural dentin matrix products by three isoforms of bone morphogenetic protein-1 (BMP1). *Matrix Biology*.

[4] Yamakoshi Y, Hu JCC, Iwata T, Kobayashi K, Fukae M, Simmer JP (2006). Dentin sialophosphoprotein is processed by MMP-2 and MMP-20 in vitro and in vivo. *Journal of Biological Chemistry*.

[5] Zhang J, Wang J, Ma Y (2011). A novel splicing mutation alters DSPP transcription and leads to dentinogenesis imperfecta type II. *PLoS One*.

[6] Xiao S, Yu C, Chou X (2001). Dentinogenesis imperfecta 1 with or without progressive hearing loss is associated with distinct mutations in DSPP. *Nature Genetics*.

[7] Zhang X, Zhao J, Li C (2001). DSPP mutation in dentinogenesis imperfecta shields type II. *Nature Genetics*.

[8] Rajpar MH, Koch MJ, Davies RM, Mellody KT, Kielty CM, Dixon MJ (2002). Mutation of the signal peptide region of the bicistronic gene DSPP affects translocation to the endoplasmic reticulum and results in defective dentine biomineralization. *Human Molecular Genetics*.

[9] Malmgren B, Lindskog S, Elgadi A, Norgren S (2004). Clinical, histopathologic, and genetic investigation in two large families with dentinogenesis imperfecta type II. *Human Genetics*.

[10] Kim JW, Nam SH, Jang KT (2004). A novel splice acceptor mutation in the DSPP gene causing dentinogenesis imperfecta type II. *Human Genetics*.

[11] Kim JW, Hu JCC, Lee JI (2005). Mutational hot spot in the DSPP gene causing dentinogenesis imperfecta type II. *Human Genetics*.

[12] Lee SK, Lee KE, Jeon D (2009). A novel mutation in the DSPP gene associated with dentinogenesis imperfecta type II. *Journal of Dental Research*.

[13] Lee SK, Hu JCC, Lee KE, Simmer JP, Kim JW (2008). A Dentin Sialophosphoprotein Mutation That Partially Disrupts a Splice Acceptor Site Causes Type II Dentin Dysplasia. *Journal of Endodontics*.

[14] Hart PS, Hart TC (2007). Disorders of human dentin. *Cells Tissues Organs*.

[15] Zhang X, Chen L, Liu J (2007). A novel DSPP mutation is associated with type II dentinogenesis Imperfecta in a chinese family. *BMC Medical Genetics*.

[16] Holappa H, Nieminen P, Tolva L, Lukinmaa PL, Alaluusua S (2006). Splicing site mutations in dentin sialophosphoprotein causing dentinogenesis imperfecta type II. *European Journal of Oral Sciences*.

[17] Wang H, Hou Y, Cui Y (2009). A novel splice site mutation in the dentin sialophosphoprotein gene in a Chinese family with dentinogenesis imperfecta type II. *Mutation Research*.

[18] Song Y, Wang C, Peng B (2006). Phenotypes and genotypes in 2 DGI families with different DSPP mutations. *Oral Surgery, Oral Medicine, Oral Pathology, Oral Radiology and Endodontology*.

[19] Song YL, Wang CN, Fan MW, Su B, Bian Z (2008). Dentin phosphoprotein frameshift mutations in hereditary dentin disorders and their variation patterns in normal human population. *Journal of Medical Genetics*.

[20] Kida M, Tsutsumi T, Shindoh M, Ikeda H, Ariga T (2009). De novo mutation in the DSPP gene associated with dentinogenesis imperfecta type II in a Japanese family. *European Journal of Oral Sciences*.

[21] McKnight DA, Hart PS, Hart TC (2008). A comprehensive analysis of normal variation and disease-causing mutations in the human DSPP gene. *Human Mutation*.

[22] McKnight DA, Simmer JP, Hart PS, Hart TC, Fisher LW (2008). Overlapping DSPP mutations cause dentin dysplasia and dentinogenesis imperfecta. *Journal of Dental Research*.

[23] Bai H, Agula H, Wu Q (2010). A novel DSPP mutation causes dentinogenesis imperfecta type II in a large Mongolian family. *BMC Medical Genetics*.

[24] Nieminen P, Papagiannoulis-Lascarides L, Waltimo-Siren J (2011). Frameshift mutations in dentin phosphoprotein and dependence of dentin disease phenotype on mutation location. *Journal of Bone and Mineral Research*.

[25] Li D, Du X, Zhang R (2012). Mutation identification of the DSPP in a Chinese family with DGI-II and an up-to-date bioinformatic analysis. *Genomics.*.

[26] Ye L, MacDougall M, Zhang S (2004). Deletion of Dentin Matrix Protein-1 Leads to a Partial Failure of Maturation of Predentin into Dentin, Hypomineralization, and Expanded Cavities of Pulp and Root Canal during Postnatal Tooth Development. *Journal of Biological Chemistry*.

[27] von Marschall Z, Mok S, Phillips MD, McKnight DA, Fisher LW (2012). Rough endoplasmic reticulum trafficking errors by different classes of mutant dentin sialophosphoprotein (DSPP) cause dominant negative effects in both dentinogenesis imperfecta and dentin dysplasia by entrapping normal DSPP. *Journal of Bone and Mineral Research*.

[28] Tsukumo Y, Tsukahara S, Saito S, Tsuruo T, Tomida A (2009). A novel endoplasmic reticulum export signal: proline at the +2-position from the signal peptide cleavage site. *Journal of Biological Chemistry*.

